# A potential antifungal bioproduct for *Microsporum canis*: Bee venom

**DOI:** 10.4102/ojvr.v91i1.2191

**Published:** 2024-12-17

**Authors:** Armağan E. Ütük, Tülin Güven Gökmen, Hatice Yazgan, Funda Eşki, Nevin Turut, Şifa Karahan, İbrahim Kıvrak, Sedat Sevin, Osman Sezer

**Affiliations:** 1 Department of Parasitology, Ceyhan Veterinary Faculty, Cukurova University, Adana, Türkiye; 2 Department of Biotechnology, Institute of Natural and Applied Sciences, Cukurova University, Adana, Türkiye; 3 Department of Microbiology, Ceyhan Veterinary Faculty, Cukurova University, Adana, Türkiye; 4 Department of Food Hygiene and Technology, Ceyhan Veterinary Faculty, Cukurova University, Adana, Türkiye; 5 Department of Obstetrics and Gynecology, Ceyhan Veterinary Faculty, Cukurova University, Adana, Türkiye; 6 Bacteriology Laboratory, Adana Veterinary Control Institute, Adana, Türkiye; 7 Department of Chemistry and Chemical Processing Technologies, Cosmetic Technology Program, Muğla Vocational School, Muğla Sıtkı Koçman University, Muğla, Türkiye; 8 Department of Pharmacology and Toxicology, Veterinary Faculty, Ankara University, Ankara, Türkiye; 9 Parasitology Laboratory, Adana Veterinary Control Institute, Adana, Türkiye

**Keywords:** bee venom, *Microsporum canis*, antifungal activity, broth dilution method, bioproduct

## Abstract

Natural treatment options for *Microsporum canis* dermatophytosis are being explored because of resistance to several antifungal medications. In this study, the potential antifungal effect of bee venom (BV), a natural antimicrobial agent, on *M. canis* was investigated. The antifungal effects of BV, fluconazole, itraconazole, amphotericin B and terbinafine were evaluated by the macrodilution method at various concentrations by modifying the microdilution method recommended by the European Committee on Antimicrobial Susceptibility Testing. All isolates were observed to be susceptible to terbinafine and fully resistant to fluconazole and amphotericin B. Minimum inhibitory concentration (MIC) and minimum fungicidal concentration (MFC) values of *M. canis* isolate 2 (Mc2) were determined as 8 µg/mL for itraconazole. The MIC and MFC values of BV were found to be 320 µg/mL for the Mc2 isolate and 640 µg/mL for the Mc6 isolate. The results showed that the isolates obtained from clinical samples in this study were highly resistant to all antifungal agents, except terbinafine. The increase in resistance indicates that antifungal drugs will become insufficient and ineffective over time and natural products such as BV should be evaluated as alternatives.

**Contribution:**

Although there are many drugs for the treatment of *M. canis*, the increase in resistance to antifungal agents reveals the need for the identification and development of new natural agents. Bee venom, which has been shown to have a safe and weak allergenic effect in various studies, can be tested for usability as a local antifungal drug when supported by *in vivo* studies.

## Introduction

*Microsporum canis (M. canis)* is a dermatophyte that is common in cats and dogs and can cause small epidemics in society because of its zoonotic potential. *Microsporum canis* is responsible for 90% of feline dermatophytoses worldwide (Frymus et al. 2013). Arthrospores can remain infectious for about 18 months. The contaminant is transmitted to humans through fomite or direct contact with animals, causing severe superficial mycosis. Although it is possible to vaccinate animals from 2 months of age, the vaccine cannot induce a sufficient immune response to protect against *M. canis* (Sparkes et al. 1994).

Treatment in humans and animals is topical or systemic, depending on the clinical situation. The main antifungal agents used in the fight against mycosis in cats and dogs are azole antifungals (ketoconazole, enilconazole, itraconazole, miconazole, clotrimazole, econazole, thiabendazole, fluconazole), allylamines (terbinafine), polyenes (amphotericin B, nystatin, natamysin), griseofulvin, lufenurondes (De Pauw 2000). In oral therapies, ravuconazole, and in topical applications, luliconazole are among the new treatment options (Kano et al. 2023). Drugs such as griseofulvin, terbinafine, itraconazole and fluconazole are used to treat severe infections in humans and animals (Aneke, Otranto & Cafarchia 2018). Recently, successful results have been obtained in animals through biological control with preparations containing *Pythium oligandrum.*

In recent studies, it has been reported that in 25% – 40% of treated patients, treatment was unsuccessful and even relapsed. Reasons for failure to achieve treatment success include insufficient penetration of the drug into the tissue, variability in drug bioavailability and resistance of the dermatophyte to the drug. Due to the development of resistance to fluconazole and itraconazole used in the treatment of dermatophytosis and also the side effects of frequently used terbinafine, has increased interest in antifungal natural product options. The antifungal activity of bee venom (BV) has been previously tested on *Trichophyton rubrum* and *Trichophyton mentagrophytes,* and the activity at certain concentrations has been determined (Park et al. 2018; Yu et al. 2012). However, there is no study on the effect of BV on *M. canis*.

Bee venom, which is among the top 10 most powerful venoms in the world, has anti-rheumatic, anti-inflammatory, anti-cancer and antimicrobial activities, and is used in many areas under the name of venom therapy. Its antiviral activity has been detected in herpes simplex virus (HSV), influenza A virus, vesicular stomatitis virus (VSV), respiratory syncytial virus (RSV), enterovirus-71 and coxsackie virus (Uddin, Lee & Nikapitiya 2016). Its antibacterial activity has also been shown on many pathogens and even multidrug-resistant nosocomial infection agents (Gökmen et al. 2023a, 2023b). The antifungal activity for *Candida spp., Malessezia spp., Aspergillus spp.* and *Trichophyton spp.* has also been investigated in various studies (Lee 2016; Lee & Lee 2010; Yu et al. 2012). However, studies have not been found on *M. canis*, which causes current and persistent dermatophytosis in animals and humans and should be followed up because of drug resistance seen in recent years.

However, there are always open questions about the potential toxicity of BV, which often shows good results against many diseases, against normal non-target cells and tissues. Toxicity is one of the biggest obstacles to its use in therapy. Bee venom is also known to have cytotoxic and genotoxic properties, to damage deoxyribonucleic acid (DNA) structure and cell membranes, and to have lytic properties when embedded in phospholipid layers. Current research data support the possibility of using such animal-derived venoms in the development of therapeutic agents (Bordon et al. 2020; Lima & De Lima 2023). However, the route of injection, molecular target, mechanism of action, exact dosage, possible side effects and other basic parameters should be investigated further before their possible clinical use (Garaj-Vrhovac & Gajski 2009; Sjakste & Gajski et al. 2023).

Our study aimed to examine the effectiveness of BV, one of the most potent antimicrobial agents in nature, on *M. canis*, the most important factor in zoonotic dermatophytosis and compare to the antifungal drugs itraconazole, fluconazole, amphotericin B and terbinafine.

## Research methods and design

### Obtaining bee venom and determining contents

Bee venom samples were obtained from Apis mellifera Anatolia (Muğla ecotype) hives in Menteşe, Muğla, Türkiye provinces during the citrus honey season between May 2021 and June 2021. Samples were collected using a BV collector (BeeSas Apiculture, Turkey). Bee venom was freeze-dried and stored in a freezer at −18 °C until analysed (Gökmen et al. 2023b).

### Sample collection

Clinical samples were taken from 17 cats who were brought to different veterinary clinics with suspicion of dermatophytosis and with a microscopic diagnosis of 10% potassium hydroxide (KOH) and examined with wood light. Cats included in the study were selected from those that had not previously been treated with antifungal drugs. Samples were collected from the boundary area between the injured skin and healthy skin. Before sampling, the area was disinfected with 70% alcohol. The scalpels and forceps used were sterile, and samples of the skin scraping and hair were transferred to the laboratory on sterile paper.

### Isolation and identification of *Microsporum canis*

Skin scraping and hair samples were inoculated on Saboraud Dextrose Agar (SDA) with cycloheximide (0.05%) (Merck, Darmstadt, Germany) and chloramphenicol (0.005%) (Merk, Darmstadt, Germany), and Potato Dextrose Agar (PDA). Plates were then incubated for about 10–14 days at 28 °C. At the end of the second week, *M. canis* was identified by its macro and microscopic features (George 2017).

### Susceptibility test

For macrodilution method, itraconazole, fluconazole, terbinafine, and amphotericin B (Merck, Damstadt, Germany) were dissolved in 100% dimethyl sulfoxide (DMSO) (Gibco) according to the National Committee for Clinical Laboratory Standards (NCCLS) protocol, and 1000 µg/mL stock solutions were prepared. Then, antifungal stock solutions were diluted with Saboraud Glucose Broth (SGB) and eight different concentration tubes were prepared (Table 1). The *M. canis* inoculum in each tube was adjusted as 1–2.5 × 10^5^ cfu/mL. It was incubated at 28 °C for approximately 10–12 days to determine the minimum inhibitory concentration (MIC) value. Then, the minimum fungicidal concentration (MFC) values were determined by inoculating the samples in the tubes on the SDA. All tests were performed in triplicate.

**TABLE 1:** Sensitivity of *M. canis* isolates to antifungal drugs and bee venom concentrations.

Isolates	Fluconazole								Itraconazole								Terbinafine								Amphotericin B								Bee venom							
	1 µg/mL	2 µg/mL	4 µg/mL	8 µg/mL	16 µg/mL	32 µg/mL	64 µg/mL	128 µg/mL	0.1 µg/mL	0.25 µg/mL	0.5 µg/mL	1 µg/mL	2 µg/mL	4 µg/mL	8 µg/mL	16 µg/mL	0.1 µg/mL	0.25 µg/mL	0.5 µg/mL	1 µg/mL	2 µg/mL	4 µg/mL	8 µg/mL	16 µg/mL	0.1 µg/mL	0.25 µg/mL	0.5 µg/mL	1 µg/mL	2 µg/mL	4 µg/mL	8 µg/mL	16 µg/mL	20 µg/mL	40 µg/mL	80 µg/mL	160 µg/mL	320 µg/mL	640 µg/mL	1280 µg/mL	2560 µg/mL
Mc1	R	R	R	R	R	R	R	R	R	R	R	R	R	R	R	R	S	S	S	S	S	S	S	S	R	R	R	R	R	R	R	R	R	R	R	R	R	R	R	R
Mc2	R	R	R	R	R	R	R	R	R	R	R	R	R	R	S	S	S	S	S	S	S	S	S	S	R	R	R	R	R	R	R	R	R	R	R	R	S	S	S	S
Mc3	R	R	R	R	R	R	R	R	R	R	R	R	R	R	R	R	S	S	S	S	S	S	S	S	R	R	R	R	R	R	R	R	R	R	R	R	R	R	R	R
Mc4	R	R	R	R	R	R	R	R	R	R	R	R	R	R	R	R	S	S	S	S	S	S	S	S	R	R	R	R	R	R	R	R	R	R	R	R	R	R	R	R
Mc5	R	R	R	R	R	R	R	R	R	R	R	R	R	R	R	R	S	S	S	S	S	S	S	S	R	R	R	R	R	R	R	R	R	R	R	R	R	R	R	R
Mc6	R	R	R	R	R	R	R	R	R	R	R	R	R	R	R	R	S	S	S	S	S	S	S	S	R	R	R	R	R	R	R	R	R	R	R	R	R	S	S	S

S, sensitive; R, resistant; Mc, *Microsporum canis*.

### Antifungal activity of the bee venom against *Microsporum canis*

The effectiveness of BV on *M. canis* was determined by the macrodilution method, by modifying the method recommended by the European Committee on Antimicrobial Susceptibility Testing (EUCAST) (Arendrup et al. 2021). First of all, Bee venom was dissolved with distilled water. Bee venom stock solutions were diluted with Saboraud Glucose Broth (SGB) and eight different concentration tubes were prepared (Table 1). *M. canis* isolates were obtained as a pure colony on SDA. Thereafter, it was aimed to sporulate the isolates on PDA to produce conidia intensively and were incubated at 28 °C for approximately 10–12 days.

Then, fungal colonies were collected with cotton swabs and suspended in 5 mL of sterile saline (0.9%) containing 0.1% Tween 20 and gently shaken with a vortex and Pasteur pipette until the suspensions were completely homogenised. The densities of these suspensions were adjusted to a McFarland turbidity value of 0.5. This value corresponded to 2–5 × 10^6^ cfu/mL for dermatophytes. It was diluted 10 times with sterile saline and its concentration was adjusted to 2–5 × 10^5^ cfu/mL recommended by EUCAST. The *M. canis* inoculum in each SGB tube was adjusted as 1–2.5 × 10^5^ cfu/mL. It was incubated at 28 °C for approximately 10–12 days to determine the minimum inhibitory concentration (MIC) value. Then, the minimum fungicidal concentration (MFC) values were determined by inoculating the samples in the tubes on the SDA. All tests were performed in triplicate.

### Ethical considerations

This study was approved by the Ethics Board of the Adana Veterinary Control Institute on 04 April 2022 (Approval no: 04/04/2022-1/227).

## Result and discussion

### Isolation and identification of *Microsporum canis*

**FIGURE 1:**
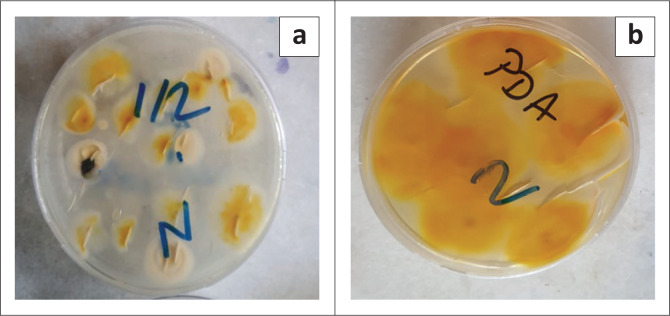
*Microsporum canis* colonies on (a) Saboraud Dextrose Agar and (b) Potato Dextrose Agar.

In our study, 17 scrapings and hair samples taken from 17 cats with suspected dermatophytosis were cultured on Saboraud Glucose Agar (SGA) and PDA media, and colonies suspicious for *M. canis* with a lemon yellow base and white hyphae were grown (Figure 1). These colonies were identified according to a previous study (George 2017). *Microsporum canis* was isolated in 6 of 17 samples (35.3%) and named as Mc1–6. *M. canis* has a high prevalence in the world, especially in temperate climate regions. In a study conducted in Northern Italy, it was reported that the prevalence of *M. canis* in street cats varies between 0.0% and 47.4% (Proverbio et al. 2014). In Japan, the prevalence of *M. canis* isolated from cats in 5 years was shown to be 25.0% (Yamada, Anzawa & Mochizuki 2019). In India and northern Brazil, the prevalence of *M. canis* was stated as 37.0% (Brilhante et al. 2003; Debnath et al. 2016).

### Susceptibility test

**FIGURE 2:**
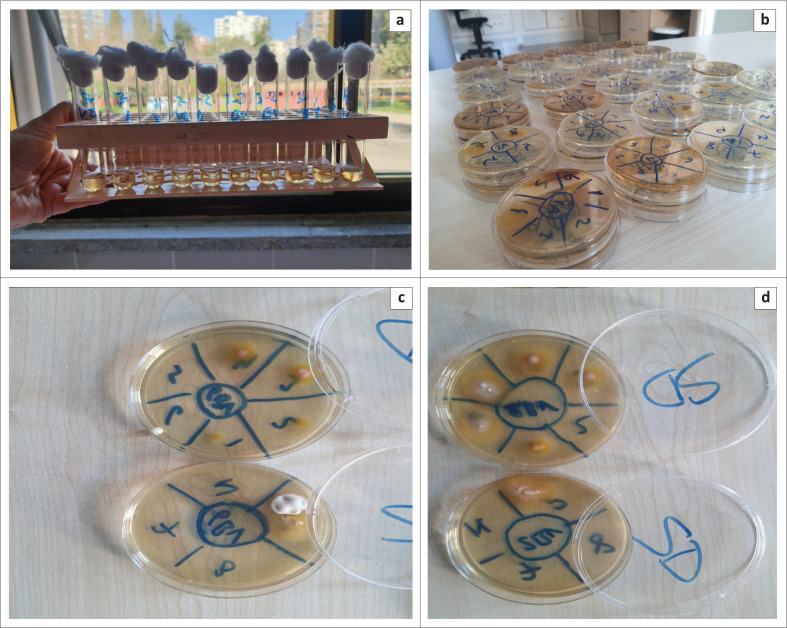
(a–d) Macrodilution method application.

The macrodilution method was applied to determine the susceptibility of *M. canis* to antifungal drugs (Figure 2). The growth turbidity of six *M. canis* isolates incubated in Sabouraud Dextrose Broth for 10 days in the tubes was examined and the concentration in the first tube with no growth was accepted as the MIC value. No growth was observed in all tubes containing terbinafine. No growth was observed in the plates when they were inoculated in SGA. The initial concentration of 0.1 µg/mL was determined as the MIC and MFC value (Table 1 and Table 2).

**TABLE 2:** Minimum inhibitory concentration and minimum fungicidal concentration values of antifungal drugs and bee venom for *M. canis* isolates.

Isolates	Fluconazole		Itraconazole		Terbinafine		Amphotericin B		Bee venom	
	MIC µg/mL	MFC µg/mL	MIC µg/mL	MFC µg/mL	MIC µg/mL	MFC µg/mL	MIC µg/mL	MFC µg/mL	MIC µg/mL	MFC µg/mL
Mc1	-	-	-	-	0.1	0.1	-	-	-	-
Mc2	-	-	8	8	0.1	0.1	-	-	320	320
Mc3	-	-	-	-	0.1	0.1	-	-	-	-
Mc4	-	-	-	-	0.1	0.1	-	-	-	-
Mc5	-	-	-	-	0.1	0.1	-	-	-	-
Mc6	-	-	-	-	0.1	0.1	-	-	640	640

MIC, minimum inhibitory concentration; MFC, minimum fungicidal concentration; Mc, *Microsporum canis*.

Although terbinafine is a keratinophilic antifungal with excellent activity against dermatophytes, terbinafine resistance has recently been observed in *M. canis* isolates (Hsiao et al. 2018). However, no terbinafine resistance was observed in our study; these results are consistent with a study from Japan where MIC values of 41 *M. canis* isolates from cats were determined to be in the range of 0.03 µg/mL – 0.25 µg/mL (Kano et al. 2023).

In the fluconazole and amphotericin B macrodilution test, growth was observed in all SGB concentrations of all isolates. Growth was observed in the plates when they were inoculated in SGA. That is, all isolates are resistant to these antifungals (Table 1 and Table 2).

Although amphotericin B is effective against *Aspergillus* and *Candida* species, *Cryptococcus neoformans, Histoplasma capsulatum, Sporothrix schenckii*, Zygomycetes, *Dematiaceous* fungi and *Fusarium* species *in vitro*, some studies have determined that it is superior to azoles and less effective than terbinafine against persistent dermatophytosis (Medoff et al. 1992; Sinha & Sardana 2018). For this reason, it was included in our study; but it was not found effective.

Fluconazole is an antifungal used to treat *M. canis* dermatophytosis in cats and humans. However, in the studies conducted in recent years, it has been reported that the *in vitro* effect of azoles, especially fluconazole, against dermatophytes isolated from animals has decreased and is now unsuccessful in the treatment of dermatophytosis (Al-Khikani & Ayit 2021). Accumulation of fluconazole in the skin and keratin is not at the same level as itraconazole and ketoconazole (Begum & Kumar 2020). In our study, it was confirmed that fluconazole had no activity on *M. canis* isolates.

In the itraconazole macrodilution test, no growth was detected in one of the isolates at 8 µg/mL and 16 µg/mL. Minimum inhibitory concentration and MFC values were accepted as 8 µg/mL. Resistance was not determined in all five other isolates (Table 1 and Table 2). In various studies, it has been observed that the MIC value varies between 0.001 µg/mL and 16 µg/mL in various countries. In a study conducted in Türkiye, the MIC value was determined as 32 in two *M. canis* isolates. This showed that there was a resistance potential in *M. canis* isolates to itraconazole, which is used effectively against dermatophytes in our country (Aktas et al. 2014; Aneke et al. 2018).

When the macrodilution test results of antifungal drugs are evaluated, it is seen that our isolates are quite resistant to other antifungals, except for terbinafine.

### Antifungal activity of the bee venom against *Microsporum canis*

The increasing resistance to antifungal drugs indicates that these drugs will become insufficient and ineffective over time and hence new equivalent products are needed. Therefore, this study aimed to investigate the antifungal effect of BV on *M. canis* using the macrodilution method. Although the effectiveness of BV on Trichophyton species has been tested in previous studies, no study on *M. canis* has been found (Park et al. 2018; Yu et al. 2012).

In our study, when BV concentrations were evaluated, the MIC and MFC value of 1 isolate out of 6 was found to be 320 µg/mL, and the other isolate was 640 µg/mL (Table 1 and Table 2). In a study conducted in South Korea, it was reported that raw BV showed statistically significant activity on *Trichophyton rubrum* at a concentration of 40 mg/100 µl. In addition, this study reported that the substances contained in BV such as apamin and melittin do not have antifungal activity alone, but raw BV shows the activity as a whole (Park et al. 2018). In another study conducted in South Korea, it was determined that the effective concentration for *Trichophyton mentagrophytes* was 0.63 ppm (parts per million), and the effective concentration for *Trichophyton rubrum* was 15–30 ppm. In the same study, it was stated that BV had a much stronger antifungal effect than fluconazole (Yu et al. 2012).

Bee venom is used therapeutically in dermatological diseases. It has been stated that it showed statistically significant effectiveness in *in vitro* and *in vivo* studies for the treatment of acne, alopecia, atopic dermatitis, morphea, psoriasis and vitiligo, and its side effects had an insignificant difference in control groups (Kim, Park & Lee 2019).

In another study, when BV was administered intradermally to male rabbits, pathological effects were not observed in blood values, liver, kidney, spleen tissues and heart muscles in histopathological, haematological and biochemical examinations. It produced a local allergic effect only at the injection site. For this reason, it was thought that it could be used as a locally and systemically safe drug (Elfiky et al. 2023).

Recent research suggests that BV has antimicrobial properties that could potentially be used in food preservation. Therefore, the use of BV as a food preservative is a relatively new concept that has attracted attention in recent years (Isidorov et al. 2023).

In a study conducted in Egypt, BV was added to traditional Talaga cheese as a food preservative and tested *in vitro*. A concentration of 15 mg/g BV was used and it was shown to be effective on *Aspergillus, Penicillium* and *Fusarium* fungi (Ahmed et al. 2024).

When all findings and data were evaluated, it was determined that although BV was not as effective as terbinafine, it showed partial antifungal activity against clinical *M. canis* isolates compared to other antifungal drugs. It is recommended that necessary *in vivo* studies be carried out to use BV, which has been found safe enough to be used as a food preservative and systemic drug in studies, as a local medicine in superficial dermatophytosis.
